# Placental Autophagy and Viral Replication Co-localize in Human and Non-human Primate Placentae Following Zika Virus Infection: Implications for Therapeutic Interventions

**DOI:** 10.3389/fviro.2021.720760

**Published:** 2021-09-17

**Authors:** Jennifer R. McKinney, Maxim D. Seferovic, Angela M. Major, Melissa A. Suter, Suzette D. Tardif, Jean L. Patterson, Eumenia C. C. Castro, Kjersti M. Aagaard

**Affiliations:** 1 Departments of Obstetrics and Gynecology, Division of Maternal-Fetal Medicine, Baylor College of Medicine and Texas Children’s Hospital, Houston, TX, United States; 2 Pathology and Laboratory Medicine, Baylor College of Medicine and Texas Children’s Hospital, Houston, TX, United States; 3 Southwest National Primate Research Center, Texas Biomedical Research Institute, San Antonio, TX, United States; 4 Department of Virology and Immunology, Texas Biomedical Research Institute, San Antonio, TX, United States; 5 Molecular and Human Genetics, Baylor College of Medicine, Houston, TX, United States

**Keywords:** autophagy, congenital Zika syndrome, placental viral infection, autophagy and viral infection, viral transmission, Zika virus

## Abstract

**Background:**

Multiple studies have shown both induction and inhibition of autophagy during Zika virus (ZIKV) infection. While some have proposed mechanisms by which autophagic dysregulation might facilitate ZIKV vertical transmission, there is a lack of *in situ* data in human and non-human primate models. This is an especially pertinent question as autophagy-inhibitors, such as hydroxychloroquine, have been proposed as potential therapeutic agents aimed at preventing vertical transmission of ZIKV and other RNA viruses.

**Objectives:**

Given the paucity of pre-clinical data in support of either autophagic enhancement or inhibition of placental ZIKV viral infection, we sought to assess cellular, spatial, and temporal associations between placental ZIKV infection and measures of autophagy in human primary cell culture and congenital infection cases, as well as an experimental non-human primate (marmoset, *Callithrix jacchus*) model.

**Study Design:**

Primary trophoblast cells were isolated from human placentae (*n* = 10) and infected *in vitro* with ZIKV. Autophagy-associated gene expression (*ULK-1, BECN1, ATG5, ATG7, ATG12, ATG16L1, MAP1LC3A, MAP1LC3B, p62/SQSTM1*) was then determined by TaqMan qPCR to determine fold-change with ZIKV-infection. In *in vivo* validation experiments, autophagy genes *LC3B* and *p62/SQSTM1* were probed using *in situ* hybridization (ISH) in the placentae of human Congenital Zika Syndrome (CZS) cases (*n* = 3) and ZIKV-infected marmoset placenta (*n* = 1) and fetal tissue (*n* = 1). Infected and uninfected villi were compared for mean density and co-localization of autophagic protein markers.

**Results:**

Studies of primary cultured human trophoblasts revealed **decreased** expression of autophagy genes *ATG5* and *p62/SQSTM1* in ZIKV-infected trophoblasts [*ATG5* fold change (±SD) 0.734-fold (±0.722), *p* = 0.036; p62/SQSTM1 0.661-fold (±0.666), *p* = 0.029]. Histologic examination by ISH and immunohistochemistry confirmed spatial association of autophagy and ZIKV infection in human congenital infection cases, as well as marmoset placental and fetal tissue samples. When quantified by densitometric data, autophagic protein LC3B, and p62/SQSTM1 expression in marmoset placenta were significantly *decreased* in *in situ* ZIKV-infected villi compared to less-infected areas [LC3B mean 0.951 (95% CI, 0.930–0.971), *p* = 0.018; p62/SQSTM1 mean 0.863 (95% CI, 0.810–0.916), *p* = 0.024].

**Conclusion:**

In the current study, we observed that in the non-transformed human and non-human primate placenta, disruption (specifically down-regulation) of autophagy accompanies later ZIKV replication *in vitro, in vivo*, and *in situ*. The findings collectively suggest that dysregulated autophagy spatially and temporally accompanies placental ZIKV replication, providing the first *in situ* evidence in relevant primate pre-clinical and clinical models for the importance of timing of human therapeutic strategies aimed at agonizing/antagonizing autophagy. These studies have likely further implications for other congenitally transmitted viruses, particularly the RNA viruses, given the ubiquitous nature of autophagic disruption and dysregulation in host responses to viral infection during pregnancy.

## INTRODUCTION

Autophagy is a critical cytoplasmic process of degradation, which is felt to be crucial in the maintenance of cellular and tissue integrity. It occurs at a basal level in eukaryotic cells, in which cytoplasmic contents are partitioned into a double membrane-enclosed autophagosome (AP). This AP complex can then fuse with lysosomes to participate in cellular degradation and recycling. Every step of the process (initiation, vesicle elongation, maturation, docking and fusion, vesicle degradation) is controlled by a complex set of regulatory proteins (1–3). Over the past decade, interest in the role of autophagy in many areas of the pathophysiology of human disease has increased, including cellular response to infection.

There are several examples of co-evolution of viral mechanisms that take advantage of (or co-opt) host clearance mechanisms to accentuate their virulence. For example, recognition of viral antigens and the subsequent triggering of interferons and neutralizing antibodies limit viral replication, and thus viruses have co-opted key aspects of immune recognition and interferon production to instead facilitate their replication with enhanced cell entry and suppressed immunoreactivity (1, 4). Autophagic mechanisms that clear viruses, as obligate intracellular organisms, are no exception. Japanese encephalitis (5), HIV (6), Dengue (7, 8), Ebola (9), Parvo, Polio, Coxsackie, Hepatitis B, and Hepatitis C (10) viruses are all examples of viruses that exploit autophagy to enhance their replication and trigger host pathogenesis in the process (11).

Zika virus (ZIKV), a positive-strand RNA arbovirus in the *Flaviviridae* family initially identified in Uganda in 1947, evolved an Asian clade that then was identified as causing birth anomalies after emerging in the Caribbean and South America in late 2014 and early 2015 (12, 13). The latest pathological Asian strain has since become endemic, not only regionally, but has re-emerged in Africa and Asia in a largely immuno-naïve population (14). Recognition of congenital Zika syndrome (CZS), a phenotype that includes severe fetal neural anomalies (12, 15) during the last epidemic in South America, spurred a wave of research into mechanism and route of ZIKV infection and perinatal transmission (13, 16–23). Through this research, autophagy was identified as a potential key interrupted process rendering CZS. This was not entirely surprising, as dysregulation of autophagy has been previously linked to neural pathogenesis in neurodegenerative conditions (24), is known to be important for normal neurogenesis in human fetal neural stem cells (25, 26), and has been shown to affect central nervous system consequences of ZIKV infection in a pregnant mouse model (27). Moreover, *in vitro* infection models demonstrate that some degree of dysregulation of autophagy accompanied ZIKV infection of cell lines, including human umbilical vein endothelial cells (28), fetal neural stem cells (25, 29), and human skin fibroblasts (30). Several potential genes and pathways involved in the process have been identified in non-human or primate models (29, 31–35). However, the exact manner of and mechanisms by which autophagy dysregulation might facilitate ZIKV vertical transmission is still under investigation, especially regarding avoidance of clearance through the normal anti-viral autophagic processes after initial induction. This is crucial, as pharmacologic autophagy inhibitors are both available and have historical use with safety data for other indications in pregnancy (36–38). Specifically, anti-autophagic drugs such as mefloquine (37) or chloroquine (38, 39) have been proposed as potentially effective treatment to prevent maternal-fetal transmission in ZIKV-exposed pregnancies.

Autophagic processes have previously been shown to affect resistance to infection in human placentae [for example Cao et al. (40)]. Biologically plausible candidates for autophagy dysregulation facilitating vertical transmission include several of the cells of the human placenta. Indeed, we and others previously demonstrated *in vivo, in vitro*, and *in situ* replication of ZIKV in primary human and primate placental cells (cyto- and syncytiotrophoblasts, Hofbauer cells, endothelial cells, fibroblasts) (13, 19), as well as cells in the chorioamniotic membrane (epithelial cells and trophoblast progenitors) (41). We demonstrated previously that even small focal infections of the villous tissue are sufficient for vertical transmission of the virus, even with asymptomatic maternal infection (16). Experimental infections in animals (primates and manipulated murine models) demonstrated that placental trophoblast replication preceded fetal infection (18, 42). Collectively, these data document varying degrees of placental cellular infection in both the absence and presence of fetal infection and pathogenesis, leading to the conclusion that the placenta potentially seeds and serves as a reservoir for subsequent diffuse fetal infection.

These observations inspired our current study. Because the placenta is a highly active in autophagic flux throughout pregnancy (43), we and a few others have hypothesized that the interaction of autophagy and ZIKV infection in the placenta may contribute to pathogenesis. For example, impairment of autophagy through use of knock out mouse models reduces vertical transmission and fetal and placental damage following ZIKV infection (40). Results were echoed in studies of human neuronal precursors and glial cells in culture in which ZIKV seems to induce autophagy early on during infection, but then downregulates autophagy through induction of mTOR in order to facilitate ZIKV replication (44).

Given the paucity of human clinical (observational cases) or pre-clinical experimental animal data in support of either autophagic enhancement or inhibition of placental viral infection, we sought to leverage our previous human translation and non-human experimental infection research to assess cellular, spatial, and temporal associations between placental ZIKV replication and measures of macroautophagy. The objectives were therefore to first directly assess autophagic gene expression changes in primary syncytiotrophoblast cell culture infected *in vitro*. We then aimed to provide detailed histopathology *in vivo* employing human placental tissue and our non-human primate model (*Callithrix jacchus*, common marmoset) that, unlike other mammalian models, is naturally susceptible to infection and vertical viral transmission.

## MATERIALS AND METHODS

### ZIKV-Associated Autophagy Gene Expression Changes in Primary Human Trophoblasts by qPCR

Placental donors were comprised of primarily white Hispanic women with uncomplicated term deliveries, without major maternal or fetal comorbidities or anomalies, and high integrity placental tissue samples. Subjects and their samples were recruited via methods described previously in the index study for the current report (13). The clinical characteristics of the donor pregnancies are summarized in Supplementary Table 1. Briefly, placental tissue was dissected, then enzymatically digested, then Percoll separated to isolate cytotrophoblasts, which then syncytialized *in vitro*. After 2–5 days, syncytiotrophoblasts were infected with 1 × 10^5^ RNA copies of first passage clinically isolated ZIKV FLR [10 Tissue Culture Infectious Dose (TCID) or mock]. Active replication was confirmed as described in our previously published work (13). After 4–5 days cells were scraped and flash-frozen in RNA*later* (ThermoFisher Scientific). Exosomal RNA was isolated from ZIKV-infected trophoblast cultures using TRIzol LS (Invitrogen) purification as previously described (13). Levels of specific mRNAs for autophagy-associated genes (*ULK1, BECN1, ATG5, ATG7, ATG12, ATG16L1, MAP1LC3A, MAP1LC3B, p62/SQSTM1*) were assessed against GAPDH as the endogenous control, using TaqMan qPCR assays (ThermoFisher Scientific) (assay IDs are listed in Supplementary Table 2). Template cDNA was generated using High Capacity cDNA reverse transcription Kit (Applied Biosystems) per manufacturer’s instructions. Triplicate reactions were then prepared using 1 ug template cDNA per well. TaqMan assays were performed with a StepOnePlus platform (Applied Biosystems) according to standard conditions of 60°C annealing temperature for 40 cycles. Delta *C_t_* data was filtered for outliers with a *Q* = 0.01. Differences were determined using paired *t*-tests within donors compared to mock. Fold change was calculated by delta delta *C_t_* method. All statistics were performed with Prism software (GraphPad, v8.4.3, La Jolla, CA).

### Co-localization of ZIKV Replication and Autophagy Activity in Human and Marmoset Placentae via *In situ* Hybridization and Immunohistochemistry

No new samples were obtained for this current work. Histological sections from human case reports as well as experimental non-human primate infections were obtained from two separate studies that have been previously published (16, 19) [human studies were approved by Baylor College of Medicine IRB H-25735; non-human primate studies were performed at Southwest National Primate Research Center and approved by local Institutional Animal Care and Use Committee (IACUC) and Biohazard Committee]. For the human study, subjects with risk for, or suspected, CZS were recruited and consented as described in the previous publication (16). A clinical description of the cases is summarized in Supplementary Table 3. Immediately following delivery, a full cross section of placental tissue was removed 4 cm from the cord insertion site for three suspected CZS cases, and one healthy neonate. Tissue was then further dissected and fixed in formalin for ~8 h then processed into paraffin blocks.

Under IACUC approval, two pregnant common marmosets were experimentally infected with an injection of 2.5 × 10^5^ plaque forming units (PFU) of the first passage contemporaneous Brazil ZIKV strain SPH2015 (GenBank accession number KU321639) twice 4 days apart, on estimated gestational days 79/83 and 68/72, respectively, as previously published (19). After 16 days (dam 1) and 18 days (dam 2) of asymptomatic infection, both pregnancies spontaneously aborted, from which tissue was obtained and formalin fixed prior to processing. Because of tissue destruction prior to initial collection, sections of collected tissue from only dam 2 were paraffin-embedded into blocks and used for the current work: marmoset fetus [full sagittal (*n* = 1) and frontal/coronal (*n* = 1)] and placenta (*n* = 1). Detailed examination of the tissues as previously described revealed focal infection and viral replication in diverse placental and fetal tissues.

In the current study, for all tissues, serial sections were meticulously prepared in parallel to allow for cross-comparison using different stains to label viral replication and markers of autophagy. To assess for viral infection, *in situ* hybridization (ISH) was carried out against the ZIKV positive strand viral genome using a ZIKV-specific probe set largely according to manufacturer’s instructions. An additional pretreatment with Protease Plus for 15min and Target Retrieval Reagent for 15min (RNAscope, ACD Biosciences) was performed for optimal retrieval. To assess for the levels of autophagy-associated proteins LC3B and p62/SQSTM1, immunohistochemistry was employed. Serial sections of the human placental blocks were deparaffinized and rehydrated, then probed with antibodies against LC3B (#3868, Cell Signaling Technology, Danvers, MA, USA) or p62/SQSTM1 (#H00008878-M01, Abnova, Walnut, CA, USA). It was determined that these were non-cross reacting antibodies with testing, and therefore LC3B (NB100-2220) and p62/SQSTM1(NBP1-49956) (Novus, Centennial, CO, USA) were alternatively used for non-human primate tissue sections.

Bright field slides were examined under low (4x), medium (20x), medium-high (40x), and high power (60x) using a Nikon Eclipse 90i microscope (Nikon Instruments, Melville, NY, USA). As a first pass, areas of active ZIKV infection were identified and marked. The same villi and areas were then identified on each serial immunohistochemistry section by comparing those markings and confirmed by comparing landmarks within the microanatomy of those areas. In this way, precise co-localization could be confidently determined. Images were captured using NIS Elements 4.20 (Nikon). Staining within areas of interest was assessed visually and using metrics within the software as described below. Minor adjustment for contrast and background levels were made using NIS Elements 4.20 for publication (Nikon).

### Quantitative Measures of Autophagy Activity in Marmoset Placentae

Sections were inspected for focal areas of infected villi. Four such areas per slide in the marmoset placental tissue were selected, where heavily ZIKV-infected villi were adjacent to apparently un-stained, and uninfected villi. Densitometry was then performed within these villi to establish and compare the levels of autophagic marker proteins. Standard colorimetric thresholds were established for each of the three stains (ZIKV, LC3B, and p62 antibodies) using NIS Elements 4.20 (Nikon). To do this, the most densely stained area of the highest power image was selected, and serial areas of staining were selected until all apparent staining was captured as assessed visually. Villi of interest were then manually traced using the inside of the syncytiotrophoblast as the perimeter. From the ZIKV stained slides, densitometry was used to systematically establish the placental villi within the captured fields with the most (*n* = 2) and least (*n* = 2) ZIKV infection. The most and least infected villi were then set as our regions of interest in the serially stained autophagy marker protein slides.

Areas were then traced to the same villi in the probed serial sections. The established thresholds for each antibody were then applied to each region of interest across all the slides, and densitometric measurements were made. Mean area density of the stain (as defined by previously-set colorimetric thresholds) was calculated for each region of interest and used as the basis of comparison. Differences between the most and least infected areas on all marmoset placental slides were determined using paired *t*-tests. All statistics were performed with Prism software (GraphPad, v8.4.3, La Jolla, CA).

## RESULTS

### Autophagy-Related Gene Expression Changes in ZIKV-Infected Primary Human Trophoblasts

To determine whether transcription of autophagic genes were altered (either up or down regulated, shown as fraction of mock-infected same subject controls) with cellular ZIKV infection, we analyzed *in vitro* infection of primary human trophoblasts. Placentas from normal, healthy term pregnancies were collected, dissected, and cytotrophoblasts isolated. *In vitro* syncytialization was monitored by assessing the βhCG levels in the conditioned media, and cell purity was assessed on a subset by subjecting the cells to flow cytometry as we have previously published (13). The syncytiotrophoblasts were then infected with first passage ZIKV FLR or a mock. The infection was confirmed by daily monitoring of the conditioned media for ZIKV RNA. After 3–5 days cells were then collected, and RNA purified to measure for gene changes of interest.

**FIGURE 1 |:**
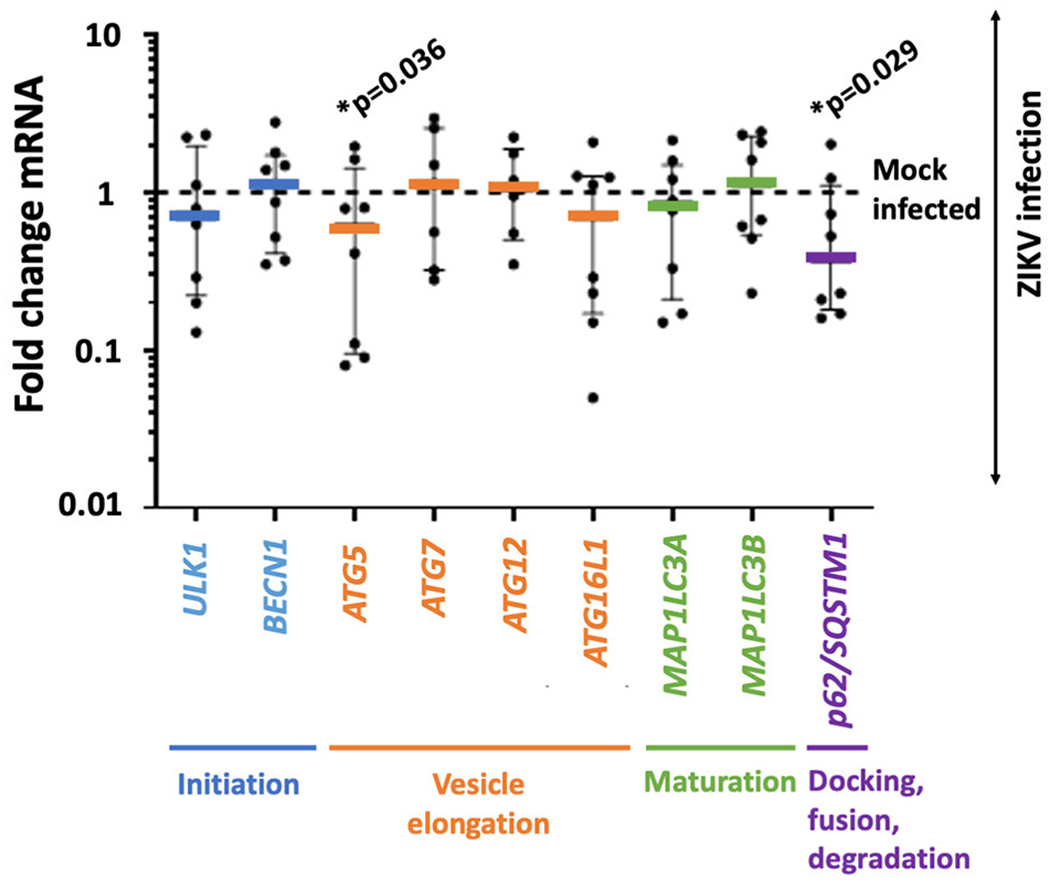
ZIKV infection is accompanied by decreased gene expression in autophagy-related genes *ATG5* and *p62/SQSTM1* in primary human trophoblasts. Primary human trophoblasts were infected with 10^5^ RNA copies of first passage clinically isolated ZIKV FLR or mock, and autophagic gene expression was assessed. Genes were selected based on involvement in multiple phases of autophagy (stage noted in colored text underlying the *x* axis). Expression of *ATG5* and *p62/SQSTM1* were significantly decreased (*ATG5* mean fold change expression 0.734 ± 0.722 SD; *p*-value 0.036, *p62/SQSTM1* mean fold change expression 0.661 ± 0.666 SD, *p*-value 0.029) following infection 2–5 days post-isolation. Fold change in expression was calculated by delta delta Ct method, normalizing first to GAPDH and then mean delta Ct of mock-infected controls. Data was filtered for outliers with *Q* = 0.01. Significance was determined using *t*-tests of *Ct*-values with a *p*-value < 0.05 denoting significance (*).

Autophagy-related genes *ULK1, BECN1, ATG5, ATG7, ATG12, ATG16L1, MAP1LC3A, MAP1LC3B*, and *p62/SQSTM1* were assessed by TaqMan qPCR. Two significant changes in gene expression were observed by *t*-test. The expression of *ATG5* was decreased by 27% (mean fold change expression 0.734 ± 0.722; *p*-value 0.036), and *p62/SQSTM1* was decreased by 33% (mean fold change expression 0.661 ± 0.666, *p*-value 0.029) compared to mock infected placental trophoblasts (Figure 1).

### Spatial Association of ZIKV Replication and Autophagy in Human Placental Tissue

Given the observed changes to key autophagy genes with *in vitro* ZIKV infection, we set out to examine known ZIKV-infected human placentae (with CZS-affected fetuses) for similar changes in protein *in situ*. Specifically, we chose LC3B as the primary marker of autophagy, as it directly interacts with our gene products of interest, as well as p62 protein. However, we first assessed the placenta for ZIKV presence via +strand RNA detection, so as to identify areas of interest with autophagic activity for examination.

**FIGURE 2 |:**
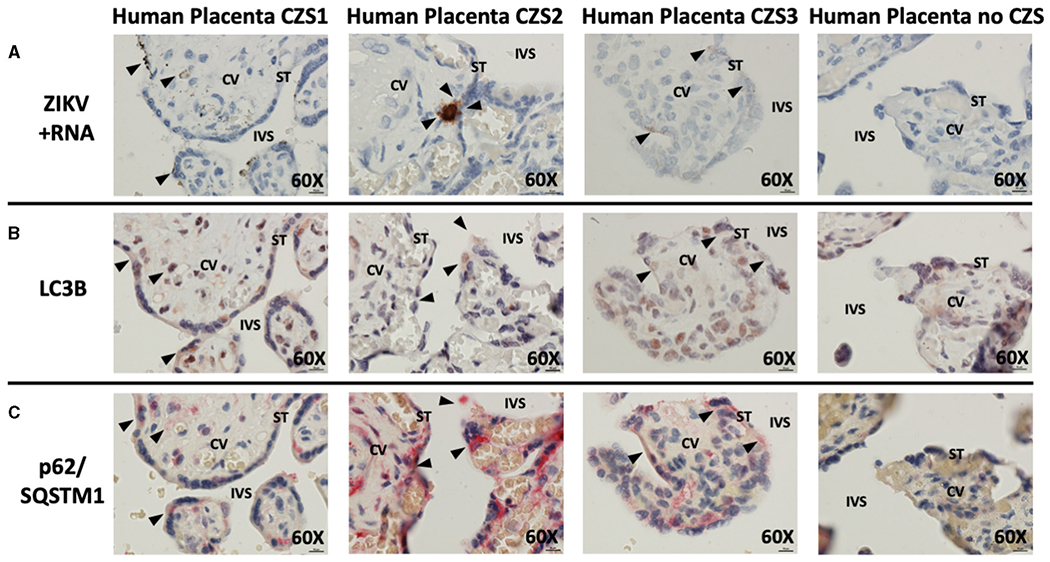
*In situ* hybridization and immunohistochemistry staining demonstrate autophagy and ZIKV replication co-localize in human placentae of neonates affected by CZS. *In situ* hybridization with positive-strand ZIKV RNA probe set demonstrates ZIKV replication within villi from confirmed CZS cases but not in the placenta from the unaffected neonate (**A**: brown labeling, black arrowheads). Immunohistochemical staining for autophagic markers LC3B (**B**: dark red-brown labeling, black arrowheads) and SQSTM1/p62 (**C**: bright red labeling, black arrowheads) was noted in the same area of the villi where ZIKV replication was present. Probe-free controls confirm no non-specific binding in the absence of ZIKV, LC3B, SQSTM1/P62 probes, respectively. All images shown were obtained at 60X magnification. Micron bar scale in bottom right corner of images = 10μm. CV, chorionic villus; IVS, intervillous space; ST, syncytiotrophoblast.

Histologic examination of cases of CZS alongside uninfected, unaffected controls demonstrated the presence of replicating ZIKV in cases but not controls (brown staining, black arrowheads, Figure 2A). The virus was noted in multiple areas of placental villi including multinucleated syncytiotrophoblasts at the maternal–fetal interface. Histologic examination of serial placental sections stained with autophagy markers LC3B and p62/SQSTM1 was performed with careful identification of exact areas of ZIKV. Co-localization of autophagy and ZIKV infection were noted in all three cases of CZS. Figure 2B demonstrates colocalization of LC3B with red-brown staining, while Figure 2C demonstrates colocalization of p62/SQSTM1 with bright red staining. Black arrowheads identify areas of ZIKV infection noted in prior sections. The small amount of visible ZIKV infection at time of tissue harvesting is likely due to the inclusion criteria of the initial study which was for infants suspected with CZS, allowing for maternal serum IgM seronegativity for ZIKV at the time of delivery.

### ZIKV and Autophagy Co-localize in Marmoset Placental Tissue as Well as Marmoset Fetal Neuro-Ophthalmic Structures

**FIGURE 3 |:**
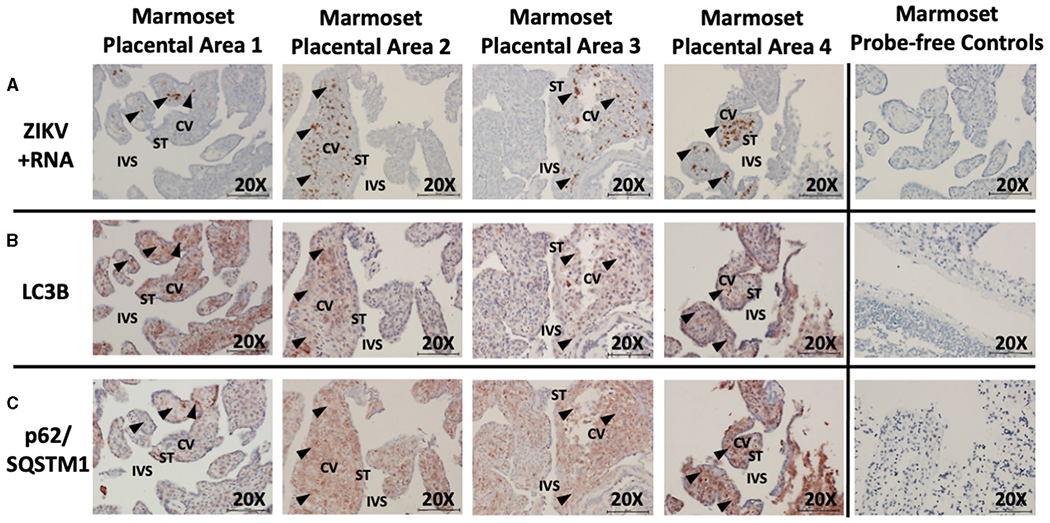
*In situ* hybridization and immunohistochemistry staining demonstrates autophagy and ZIKV replication co-localize in ZIKV-infected marmoset placenta. *In situ* hybridization with positive-strand ZIKV RNA probe set demonstrates ZIKV replication (first passage Brazil ZIKV strain SPH2015) within some villi of the placenta from a ZIKV-infected marmoset (**A**: brown labeling, black arrowheads). Immunohistochemical staining for autophagic markers LC3B (**B**: red-brown labeling, black arrowheads) and SQSTM1/p62 (**C**: red-brown labeling, black arrowheads) was noted in the same areas of the villi where ZIKV replication was present. Probe-free controls confirm no non-specific binding in the absence of ZIKV LC3B, SQSTM1/P62 probes, respectively. All images shown were obtained at 20X magnification. Micron bar scale in bottom right corner of images = 100 μm. CV, chorionic villus; IVS, intervillous space; ST, syncytiotrophoblast.

Unsurprisingly, examination of placental tissue from timed experimental infections in non-human primates showed a very high degree of ZIKV infection compared to the human cases. Dense dark brown staining was most apparent in the parenchyma of the tissue, while some staining was also apparent in the syncytiotrophoblasts. The darkest staining is most likely attributed to ZIKV infection of Hofbauer cells. Histologic examination of the placental tissue demonstrated focal areas of infection, with highly infected villi adjacent to villi without evidence of apparent ZIKV (Figure 3A, brown labeling). By comparing serial sections for morphological markers within the villous shapes, autophagy markers could be compared between infected and non-infected areas. Again, autophagy-related proteins LC3B and p62/SQSTM1 were selected as the markers of autophagy for these studies. At first glance, the staining appears universal for the constitutively expressed proteins, however co-localization within the same areas as ZIKV revealed spatial distinction (Figure 3B: LC3B, red/brown labeling; Figure 3C: p62/SQSTM1, red/brown labeling).

**FIGURE 4 |:**
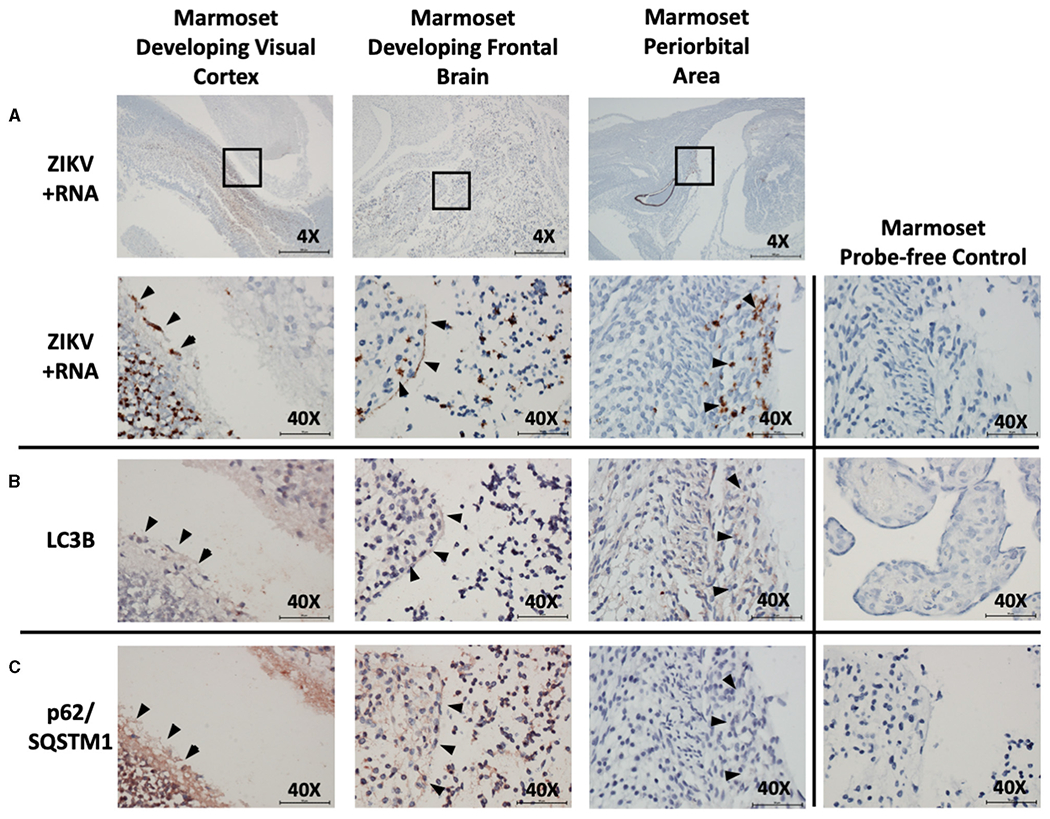
*In situ* hybridization and immunohistochemistry staining demonstrate co-localization of autophagy and ZIKV replication in neuro-ophthalmic structures of ZIKV-infected marmoset abortus. *in situ* hybridization with positive-strand ZIKV RNA probe set demonstrates ZIKV replication within the developing visual cortex, frontal brain, and periocular structures in marmoset fetus (**A**: brown labeling, black arrowheads). Boxes indicate areas imaged further at 40X. Immunohistochemical staining for autophagic markers LC3B (**B**: red-brown labeling, black arrowheads) and SQSTM1/p62 (**C**: red-brown labeling, black arrowheads) was noted in the same neuro-ophthalmic structures where ZIKV replication was present. Probe-free controls confirm no non-specific binding in the absence of ZIKV LC3B, SQSTM1/P62 probes, respectively. Images shown were obtained at 4X magnification (top row, **A**) or 40X magnification (all other images). Micron bar scale in bottom right corner of images = 500 μm (4X) or 50 μm (40X).

Examination was also made of marmoset fetal tissue from a second pregnancy with experimental infection that was spontaneously aborted. A frontal cross-section immediately posterior to the fetal eye was examined. ZIKV infection was noted within the neural tissue of the developing visual cortex, within the cortical plate of the developing frontal brain, and within the periorbital musculature. Co-localization of autophagic activity via autophagy markers LC3B (red/brown labeling) and p62/SQSTM1 (red/brown labeling) was again noted within these same fetal neuro-ophthalmic structures (Figures 4A–C). Due to the breadth of tissue types present within these sections, analysis was limited to noting co-localization of ZIKV and autophagic activity rather than allowing quantification of autophagic activity.

### Quantitative Comparison of Autophagy Protein Markers Between Infected and Uninfected Marmoset Placental Villi

**FIGURE 5 |:**
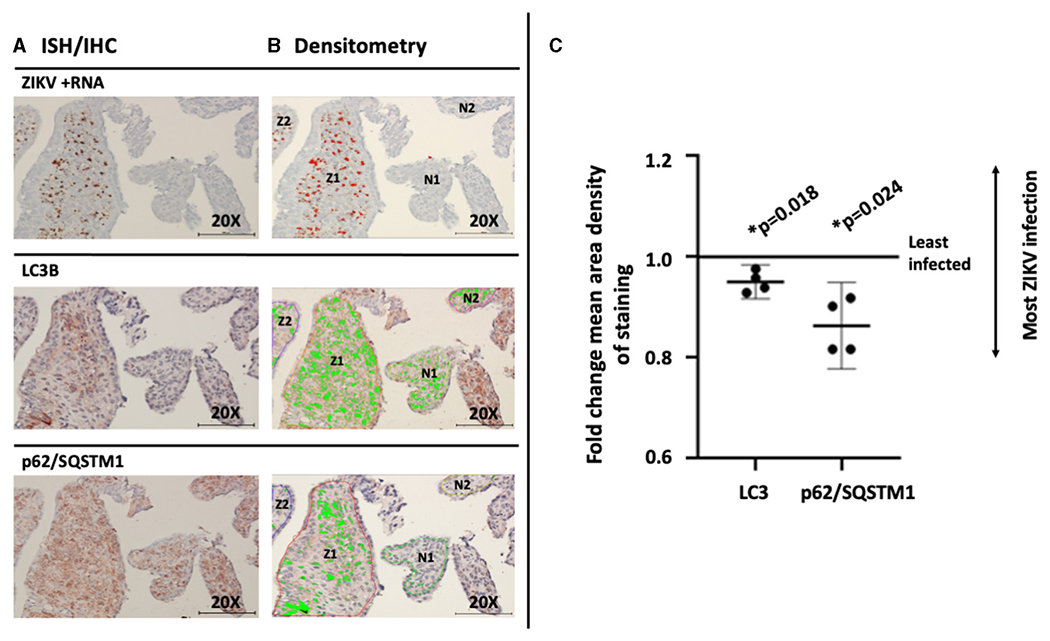
Mean density of autophagic proteins LC3B and p62/SQSTM1 is significantly decreased in the greatest compared to the least ZIKV-infected marmoset placental villi. Villi with the greatest (regions Z1–2) and least (regions N1–2) dense staining for ZIKV infection were identified using stain thresholds and defined as regions of interest **(A)**. The same greatest and least infected regions of interest were identified in subsequent sections for LC3B and p62/SQSTM1 studies, and densitometry was again used to define autophagic protein stain area in each region (**B**, bright green color). Mean area density of staining was then compared between regions of interest. Mean density of both LC3B and p62/SQSTM1 were significantly decreased in the most ZIKV-infected villi compared to the least-infected areas [**C**; LC3B mean (95%CI) 0.951 (0.930–0.971), *p* = 0.018; p62/SQSTM1 mean (95%CI) 0.863 (0.810–0.916), *p* = 0.024]. All images shown were obtained at 20X magnification. Micron bar scale in bottom right corner of images = 100 μm. Statistical significance was determined using a *p*-value < 0.05 (*).

To assess for a direct association between the viral infection and changing autophagy in an experimental *in vivo* primate model, post-imaging densitometric analysis of the bright field images was used to compare the levels of the autophagy proteins LC3B and p62/SQSTM1. Areas where infected and uninfected villous tissue was in close proximity were carefully chosen for comparison studies. Figure 5A labels ZIKV replication employing ISH. Stain thresholds were then set via post-processing densitometry to quantify the mean area of staining in each villi (defined as a region of interest) (Figure 5B). This allowed selection of villi with evidence of the most dense staining for ZIKV infection (Figures 5A,B: regions Z1–Z2) and least dense staining for ZIKV infection (Figures 5A,B: regions N1–N2). The same most ZIKV-infected and least infected villous regions of interest were identified in subsequent sections for LC3B and p62/SQSTM1 studies. Densitometry was again used to define autophagic protein stain area in each region for LC3B and p62/SQSTM1, respectively (Figure 5B, bright green color). Defined densitometric thresholds did not identify any stain in probe-free control images for ZIKV infection, LC3B, or p62/SQSTM1 (data not shown). Using the densitometric data, mean area density of staining was compared between ZIKV-infected and less-infected villi. Mean density of both LC3B and p62/SQSTM1 were significantly decreased in ZIKV-infected villi compared to less-infected areas [Figure 5C; LC3B mean (95%CI) 0.951 (0.930–0.971), *p* = 0.018; p62/SQSTM1 mean (95%CI) 0.863 (0.810–0.916), *p* = 0.024].

## DISCUSSION

### Principal Findings

In the current study, we observed that in the placenta, disruption of autophagy accompanies ZIKV replication *In vitro, In vivo*, and *In situ* in human and non-human primate infections. Specifically, we observed changes in gene expression of key autophagy genes *Atg5* and *p62/SQSTM1* accompanying ZIKV-infection and replication in human primary placental trophoblasts when compared to mock-infected controls. Similar reduction in autophagic gene expression has been shown to affect viral replication and infectivity in other models (45). When we additionally assessed measures of autophagy in experimental *in vivo* primate model and human cases of CZS, we observed significant spatial co-localization with the presence of key autophagic protein markers LC3B and p62/SQSTM1 proximal to ZIKV replication. When immunohistochemical staining in human and non-human primate placentae is quantified by densitometry, p62/SQSTM1, and to a lesser extent LC3B, are both significantly decreased in ZIKV infected placental cells when measured with an unbiased algorithmic method. These findings collectively suggest that dysregulated autophagy spatially and temporally accompanies placental ZIKV replication, providing the first *in situ* evidence in relevant pre-clinical models for human molecular therapeutic strategies aimed at agonizing/antagonizing autophagy. These studies have likely implications for other congenitally transmitted viruses, given the ubiquitous nature of autophagic disruption and dysregulation in host responses to viral infection during pregnancy.

### Results

Our findings provide key evidence which reconciles seeming disparate prior studies demonstrating ZIKV-related induction of autophagic activity and seemingly pro-viral effects of autophagy. Specifically, we demonstrate that key autophagy gene transcription and protein translation are actually significantly **decreased** in foci of ZIKV-infected normal primary (i.e., non-transformed) human and primate cells with evidence of active replication. Prior suggestions for induction of autophagy and pro-viral effect of autophagy arose from transformed (i.e., high passage placental cancer) cell lines, where a marked increase in autophagy activity was found to accompany ZIKV infection (25, 28, 30). Induction of autophagy in these transformed cell lines seems to have pro-viral effects. In addition, inhibition of autophagy, via knock-out mouse models (40) or with use of pharmacologic inhibitors (28, 30), decreases viral infection. This is in contrast to the general tenet that induction of autophagy tends to confer placental cells with high resistance to viral infection, while inhibition of autophagy confers permissivity to viral infection (46, 47). Findings supporting an anti-viral role of autophagy have been seen in human neuroprogenitor and glial cells in culture (44) and Drosophila (48), where autophagy induction is associated with restricted ZIKV infection, and inhibition is associated with promotion of ZIKV infection.

### Clinical Implications

In this current study, we demonstrate downregulation of autophagy in the endogenous setting of placental infection (e.g., *in vitro* infection of primary human trophoblasts or *in situ* measures of CZS-affected cases). Our observations reported herein show that key autophagy gene transcription and protein translation are actually significantly **decreased** in non-transformed placentae. This is further consistent with clinical observations of human cases. Namely, placental infection is not always (and even rarely) associated with either histologic nor molecular evidence of placental inflammation (49–51), but does accompany pregnancy loss and fetal infection. There may be several biological reasons for our observations.

One explanation for these seemingly contradictory findings is suggested by Sahoo et al. (44), who similarly found evidence of a temporal relationship between diminished autophagy and persistence of infection. Initial autophagy induction (with limited viral replication during a brief period of <24 h post infection) was followed by mTOR activation and subsequent down regulation of autophagy with associated increased viral replication later in the course of ZIKV infection. Initial autophagic activation may serve to provide the substrates needed for initial viral replication resulting in the decreased p62/SQSTM1 levels (signal of autophagic flux) seen in this work. Subsequent autophagic downregulation prior to viral clearance may allow continued infection and is supported by the decreased p62/SQSTM1 gene expression and decreased LC3B presence seen in our non-human primate samples.

Second, support for subsequent downregulation of autophagy with ZIKV infection also arises from experiments conducted in primary human epithelial cells, primary skin fibroblasts, and non-transformed astrocytes where an alternate pathway for ZIKV-induced cell death was identified, making it unlikely that ZIKV requires autophagy later in infection for viral spread (52). This concept is not unheard of for flaviviruses, with DENV seemingly following a similar pattern of initial induction of autophagy with primary infection, followed by inhibition of autophagy with persistent infection (7). Our results are likely most consistent with downregulation of autophagy later in course of the infection and disease process, since we observed that both the RNA from the primary human trophoblasts, and the human and non-human primate tissues were isolated past the 24 h of initial autophagy induction demonstrated by Sahoo et al. (44). p62/SQSTM1 as a pathogen recognition reception (PRR), and Atg5 which has been proposed to downregulate some PRRs, are both logical candidates for downregulation of autophagy by a virus trying to avoid normal anti-viral degradative pathways (3, 53), findings supported by this study. Put another way, if we could have sampled the same human and non-human primate placentae much earlier in infection (h), rather than days, weeks, or months later, we may have observed transient initial induction of autophagy, with subsequent downmodulation of autophagy. Such experiments will be the focus of future *in vivo* work, and our conclusions remain speculative.

### Research Implications

While this study does demonstrate spatial and temporal colocalization between autophagy and ZIKV infection at a discrete point in time, further studies are necessary to define exactly when and how autophagy is induced and/or down-modulated during the entire course of ZIKV infection, especially within human placentae. This information will better inform the timing and pharmacologic basis (if any) of potential therapeutic options. In addition, it may help identify mechanisms and pathways by which viral persistence in the placenta might occur in some, but not all, cases of congenital Zika infection and resultant fetal disease.

### Strengths and Limitations

Strengths of this study include the use of a highly relevant non-human primate model of congenital infection, with confirmation in human placental samples, both *in vitro* and *in vivo*. Limitations of this study include a small chance of cross-reactivity of the LC3B antibodies to other LC3 isoforms. In addition, the static nature of these experiments does not enable us to assess for longitudinal changes. Therefore, we cannot draw conclusions about autophagy in the initial phases of infection given our non-human primate model spontaneously aborted at 2 weeks with a highly advanced placental infection.

In general, reliance on specimens from other index studies constrained our experimental design and limited our biologic replicates. This is notably true in our *in vivo* infected marmosets (19). In the initial study, as we have previously published, we infected two pregnant marmoset dams with Brazil ZIKV at estimated gestational days 79 and 68, with a second inoculation 4 days later. As we initially detailed in the primary index study (19), spontaneous expulsion of intrauterine demised fetuses at post-inoculation day 16 (dam 1, dizygotic twins) and 18 (dam 2, singleton) occurred. The placenta from dam 1 was unavailable for analysis, as it was mauled by the dam post-delivery. The second dam’s expulsed singleton fetus and placenta were both collected and retained for analyses, and because they constituted a single exposure they were used in the current analysis (19). Recognizing the limitations of drawing meaningful conclusions based on a single pregnancies placenta and fetus, we attempted to mitigate these limitations with technical replicates by sampling different placental subsites and rigorous use of internal controls. In addition, we employed orthogonal approaches with use of human tissue specimens and primary trophoblasts in the current study. Nonetheless, we acknowledge the constraints in our primate specimens being derived from a single dam. Similarly, we acknowledge that architectural and histologic differences exist when comparing human and non-human primate placentae. However, the notable similarity of consequence for the current study is the hemochorial nature common to both human and non-human primate placentae whereby (unlike other species) the villi bathe in the maternal intervillous space. As such we feel that the marmoset is a highly appropriate model despite some anatomical differences. Moreover, we are further buoyed in the confidence of our findings and conclusions by the consistency of observations among our human and non-human primate subject’s specimens.

## CONCLUSIONS

Although the exact role of autophagy in ZIKV pathogenesis is still being determined, autophagy inhibitors, such as hydroxychloroquine (38), are being proposed as potential therapeutic agents to prevent vertical transmission and mitigate adverse fetal effects of the virus. Our results shed key light on this topic, and herald warning that such therapeutic approaches must be approached with thoughtful consideration of both timing and duration of therapy. Since our findings show that diminished autophagy later in the infection temporally and spatially co-localizes with ZIKV replication in non-human primate placentae from congenital infection cases, it cannot be concluded *de facto* that inhibition of autophagy will be of benefit. Conversely, we cannot assume that it would be of harm as our observations could be evidence of an adaptive response for maternal benefit. Rather, the appropriate interpretation and application of our findings suggest that future experimentation with anti-autophagic drugs as potentially effective in mitigating vertical transmission of ZIKV must consider the timing of administration with respect to infection. Likely subsequent downregulation of autophagic processes does not negate the benefits that such therapy may have, although does likely support the need for treatment to be limited to early in the course of infection.

## SUMMARY

Dysregulated autophagy spatially and temporally accompanies placental ZIKV replication, providing *in situ* evidence in relevant pre-clinical models for human molecular therapeutic strategies aimed at agonizing/antagonizing autophagy.

## Supplementary Material

Data Sheet

## Data Availability

The raw data supporting the conclusions of this article will be made available by the authors, without undue reservation. Requests to access these datasets should be directed to Maxim D. Seferovic, maxim.seferovic@bcm.edu.

## HIGHLIGHTS

- Given the contradictory nature of pre-clinical data supporting both autophagic enhancement and inhibition of placental ZIKV viral infection, we sought to assess cellular, spatial, and temporal associations between human and non-human primate placental ZIKV replication and measures of autophagy.
- Dysregulated autophagy spatially and temporally co-localizes with placental ZIKV replication in human placentae and non-human primate placental and fetal tissue from congenital infection cases.
- Demonstrating the relationship between dysregulated autophagy and placental ZIKV infection provides the first *in situ* evidence in relevant pre-clinical models for human therapeutic strategies aimed at agonizing/antagonizing autophagy, potentially suggesting a means for mediating vertical transmission of ZIKV.
