# Supplementary material for: Placental Autophagy and Viral Replication Co-localize in Human and Non-human Primate Placentae Following Zika Virus Infection: Implications for Therapeutic Interventions
Source: Front Virol. Author manuscript; Available in PMC 2023 Jul 10. (PMC10331925; doi:10.3389/fviro.2021.720760)
Supplement: Data Sheet [file NIHMS1913394-supplement-Data_Sheet.docx]

| **Subject ID** | **MOD** | **EGA** | **Race** | **Ethnicity** | **Comorbid conditions** | **Age** | **PP BMI** | **Medication in pregnancy** | **Infant sex** | **Post isolation DOI** |
| --- | --- | --- | --- | --- | --- | --- | --- | --- | --- | --- |
| **1** | **V** | **39w1d** | **White** | **Hispanic** | **History of PRE** | **34** | **20.2** | **PNV, aspirin** | **F** | **2** |
| **2** | **V** | **39w4d** | **Black** | **Non-Hispanic** | **None** | **26** | **NR** | **PNV** | **F** | **3** |
| **3** | **V** | **37w2d** | **White** | **Hispanic** | **None** | **27** | **32.9** | **PNV** | **F** | **3** |
| **4** | **V** | **38w3d** | **White** | **Hispanic** | **None** | **25** | **25.9** | **PNV** | **F** | **4** |
| **5** | **C** | **39w0d** | **White** | **Hispanic** | **Asthma, history of HSV** | **30** | **38.4** | **PNV, albuterol** | **F** | **4** |
| **6** | **V** | **39w3d** | **White** | **Hispanic** | **PRE** | **19** | **25.6** | **PNV** | **M** | **4** |
| **7** | **V** | **38w3d** | **White** | **Hispanic** | **History of HSV** | **33** | **23.4** | **PNV, acyclovir** | **F** | **4** |
| **8** | **C** | **39w1d** | **White** | **Hispanic** | **Oligohydramnios** | **27** | **NR** | **PNV** | **F** | **1,2,3,4,5** |
| **9** | **C** | **39w2d** | **White** | **Hispanic** | **None** | **32** | **27.4** | **PNV** | **F** | **4,5** |
| **10** | **V** | **37w1d** | **White** | **Hispanic** | **A1GDM** | **23** | **39.3** | **PNV** | **F** | **5** |
| **11** | **C** | **38w1d** | **White** | **Hispanic** | **Anemia** | **24** | **23.9** | **PNV, ferrous sulfate** | **F** | **5** |
| **12** | **C** | **39w1d** | **White** | **Hispanic** | **None** | **26** | **24.0** | **PNV** | **M** | **4** |

**Supplementary data**

**Supplementary Table 1:** Characteristics of the donors of primary placental trophoblasts used in this study. ID: subject identification number; MOD: mode of delivery; V: vaginal; C: Cesarean; GA: gestational age at delivery reported as weeks and days of gestation; A1GDM: gestational diabetes mellitus type A1; PRE: preeclampsia; PP BMI: pre-pregnancy body mass index; Post Isolation DOI: “day of infection,” specifically, what day post trophoblast isolation cells (day of collection and isolation defined as day 0) were infected with mock or ZIKV; PNV: prenatal vitamins; F: female; M: male.

| **Gene symbol** | **Gene name** | **Assay ID** | **Company** |
| --- | --- | --- | --- |
| **ULK1** | **Unc-51-like kinase 1** | **Hs00177504_m1** | **ThermoFisher Scientific** |
| **BECN1** | **Beclin 1** | **Hs01007018_m1** | **ThermoFisher Scientific** |
| **ATG5** | **Autophagy related 5** | **Hs00169468_m1** | **ThermoFisher Scientific** |
| **ATG7** | **Autophagy related 7** | **Hs00893766_m1** | **ThermoFisher Scientific** |
| **ATG12** | **Autophagy related 12** | **Hs04980076_s1** | **ThermoFisher Scientific** |
| **ATG16L1** | **Autophagy related 16 like 1** | **Hs01003142_m1** | **ThermoFisher Scientific** |
| **MAP1LC3A** | **microtubule associated protein 1 light chain 3 alpha** | **Hs01076567_g1** | **ThermoFisher Scientific** |
| **MAP1LC3B** | **microtubule associated protein 1 light chain 3 beta** | **Hs00797944_s1** | **ThermoFisher Scientific** |
| **p62/SQSTM1** | **sequestosome 1** | **Hs01061917_g1** | **ThermoFisher Scientific** |
| **GAPDH** | **glyceraldehyde-3-phosphate dehydrogenase** | **Hs02786624_g1** | **ThermoFisher Scientific** |

**Supplementary Table 2:** List of TaqMan® gene expression assays for autophagy-associated genes.

| **ID** | **Exposure risk** | **EGA at entry to US** | **Maternal symptoms**  **(EGA)** | **Prenatal US findings** | **ZIKV screening tests** | **EGA at delivery** | **Postnatal imaging and exam** | **Confirmed CZS** |
| --- | --- | --- | --- | --- | --- | --- | --- | --- |
| **1 (52)** | **Resided in endemic area** | **25** | **Rash, fever, HA, conjunctivitis**  **(20W)** | **VM, dysplastic CSP;**  **no microcephaly** | **+Serum NAT at 29, 30, 34, 35W**  **+urine NAT at 29W**  **+IgM at 30W** | **38** | **Severe VM (US, MRI),**  **FOC 54%,**  **Exam: bilateral colobomatous lesions** | **Yes** |
| **2 (65)** | **Resided in endemic area** | **11** | **none** | **VM, thin cortex, dolicocephaly;**  **no microcephaly** | **All screening tests negative** | **37** | **Colpocephaly of LV, callosal dysgenesis, right MCA vascular insult (US, MRI),**  **FOC 96%,**  **Exam: low-set ears, cleft palate, microphthalmia, asymmetric hypertonia** | **Yes** |
| **3 (237)** | **Resided in endemic area** | **15** | **none** | **MCDA twins with one demise, IC, echogenic LV walls, hypoplastic CSP;**  **Microcephaly** | **All screening tests negative** | **37** | **Severe microcephaly, supratentorial volume loss (US, MRI),**  **FOC <0.01%,**  **Exam: microcephaly** | **Yes** |
| **4 (71)** | **Resided in endemic area** | **23** | **Rash, fever**  **(21W)** | **Normal anatomy** | **+Serum NAT at 25W** | **36** | **Normal US,**  **FOC 36%,**  **Exam: normal** | **No** |

**Supplementary Table 3:** Clinical characteristics of human donors of placentae used in this study. ID: subject identification number; EGA: estimated gestational age reported as weeks of gestation; HA: headache; VM: ventriculomegaly; CSP: cavum septum pellucidum; MCDA: monochorionic diamniotic; IC: intracerebral calcifications; LV: lateral ventricles; NAT: nucleic acid amplification test; FOC: fronto-occipital (head) circumference; MCA: middle cerebral artery; CZS: Congenital Zika Syndrome
